# Research Chimpanzees May Get a Break

**DOI:** 10.1371/journal.pbio.1001291

**Published:** 2012-03-27

**Authors:** Frans B. M. de Waal

**Affiliations:** Living Links, Yerkes National Primate Research Center, Emory University, Atlanta, Georgia, United States of America

## Abstract

A recent report by the Institute of Medicine leaves few urgent reasons standing for the continued use of chimpanzees in biomedical research. It is high time to think about their retirement, Frans de Waal argues, without neglecting prospects for non-invasive research on behavior, cognition, and genetics.

When New Zealand, in 2000, became the first nation to pass legislation against research on the great apes, and Spain adopted a resolution to grant these animals legal rights, both decisions were hailed as substantial progress even though neither country conducted any actual ape research. I could not resist remarking to a Spanish journalist that I would have been more impressed had they abolished bullfighting. It is only when the Netherlands and Japan passed similar laws that the movement to improve the status of apes began to make a difference, because both countries outlawed what they had been practicing. With euthanasia ruled out as a means of population control, both governments faced the expensive need to find a home for ex-laboratory chimpanzees (*Pan troglodytes*), some of which required special precautions and care as they had been infected with HIV or hepatitis C.

The ethical grounds for this change are obvious. The same reason chimpanzees are biomedically important provides a compelling ethical argument against their use. The more an animal is like us, the easier it is to extend our moral outlook to it. Recent studies have amply documented cognitive, social, and emotional similarities between chimpanzees and humans, including empathy and the rudiments of morality, power politics, and the ability to pick up habits from each other as reflected in multiple cultural traditions across the African continent [1]–[3]. More than anyone else, Jane Goodall has impressed upon the world how deserving chimpanzees are of our protection. In every nation, thus far, authorities have mentioned the special moral standing of apes as their main reason for legislative change.

The United States has been the sole holdout. Along with the African nation of Gabon, it remains the only nation in the world with chimpanzees—nearly 1,000 of them—in biomedical facilities (Figure 1). This situation is increasingly under fire, however. The Great Ape Protection and Cost Savings Act, which would forbid all invasive biomedical research on apes, was recently reintroduced in Congress [4]. And a petition to the US Fish and Wildlife Service by the Humane Society of the United States and other groups seeks to change the status of captive chimpanzees from “threatened” to “endangered.” Under the Endangered Species Act, this upgrade would prevent commerce so that chimpanzees could not be sold for use as pets or actors in advertisements, such as the degrading CareerBuilder.com commercials [5].

**Figure 1:**
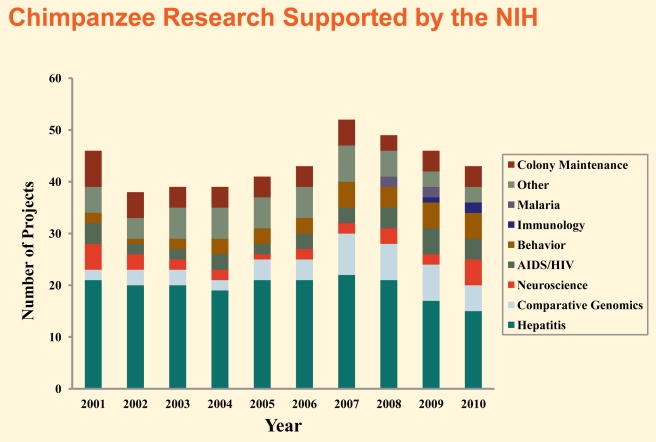
The number of annual projects funded by the NIH involving research chimpanzees has varied from 38 in 2002 to 52 in 2007. Here, the projects are broken down by topic area. From the IOM report, page 22 [7].

It is against this background that the National Institutes of Health (NIH) gave in to pressure regarding a planned move of chimpanzees from the Alamogordo Primate Facility in New Mexico to the Texas Biomedical Research Institute in San Antonio. The move would have meant a return of about 200 semi-retired chimpanzees to research. NIH asked the Institute of Medicine (IOM), an independent advisory board associated with the National Academy of Sciences, to explore how critical chimpanzees still are for biomedical research [6]. In a stark departure from debates in other nations, however, the NIH sought to exclude ethical issues from consideration, specifically declaring these issues irrelevant to the IOM's charge. This was a curious move given that the whole reason we are debating the pros and cons of research on chimpanzees, instead of rodents or other animals, is public concern about this particular species. This concern is entirely of an ethical nature. The impossibility of avoiding this issue was recognized by the IOM's appointment of a bioethicist, Dr. Jeffrey Kahn, now at Johns Hopkins University, as chairman of its committee.

The NIH's head-in-the-sand attitude towards ethics hinted that powerful interests were at play, which in turn explains the IOM's decision to keep chimpanzee experts off its committee. This way, no established interests were represented and the discussion was free from political interference. The result, though, was a committee with only tangential knowledge of the species under consideration. From the start, therefore, the committee faced serious challenges and pressures, and it is in this light that its final report is to be commended for the balance it struck and the high-quality information it delivered.

The IOM committee did an outstanding job reviewing and summarizing the biomedical need for chimpanzees in a report entitled “Chimpanzees in Biomedical and Behavioral Research: Assessing the Necessity”, released December 15, 2011 [7]. It summed up its conclusions as follows (p. 5): “The present trajectory indicates a decreasing need for chimpanzee studies due to the emergence of non-chimpanzee models and technologies.” Apart from one possible exception—on which the committee was divided—the report left few urgent reasons standing for continued biomedical use of chimpanzees. The one reason left (i.e., prophylactic hepatitis C vaccine testing) would require large numbers of chimpanzees. Since we lack such numbers, the question one half of the committee asked is what would be the point of trials with insufficient statistical power. Rodent and other rapidly developing alternative models provide sufficient immunogenicity and safety data to proceed to human efficacy trials without the need for additional chimpanzee studies. Combined with the problematic ethics of virally infecting healthy chimpanzees, I believe we now have reached the point at which NIH should take a bold stance. Reasons for continuation of current practice are becoming exceedingly tenuous. Chimpanzees may still be *useful* for biomedical research, but whether they are *critical* is in doubt. The time has come to get ahead of the “trajectory” discerned by the committee, and permanently halt all invasive research on this species.

If the NIH are not ready for such a step, they should at least convene a committee of philosophers and bioethicists to gather diverse opinions about the ethics of continuation of current practice. It is a complex issue [8],[9], and as rightly noted by the committee in a direct rebuke to the instructions it received, “any assessment of the necessity for using chimpanzees as an animal model in research raises ethical issues, and any analysis of necessity must take these ethical issues into account” (p. 2). In response to the IOM report, the NIH have announced a “working group within the NIH Council of Councils to provide advice on the implementation of the recommendations” [10]. This working group will determine which projects clear the new bar of both necessary and appropriate research, but hopefully will also include expertise to provide ethical reflection in case invasive procedures remain under consideration.

The IOM report supports the great value of chimpanzee research that is minimally harmful or painful, specifically comparative genomics, behavioral and cognitive studies, and neuroimaging. We could add postmortem tissue and brain analysis of apes that have died of natural causes. The chimpanzee (along with its congener, the bonobo, *P. paniscus*) plays a central role as touchstone of what sets us apart as a species. Knowledge of our closest living relatives also helps determine which human capacities likely have a long evolutionary history. The general trend over the last few decades has been a narrowing of the gap, bringing increasingly complex capacities under the umbrella of our primate heritage. Species-typical human characteristics remain undeniable, however, and there is a great need for continued cognitive testing and studies of genetics, neuroscience, and development to add evolutionary context to findings on human behavior. Without this kind of research, the social sciences, the humanities, and philosophy would still live in the illusion, as they did a few decades ago, that humans are totally unprecedented. Anyone who has followed the literature realizes that the vast majority of mental and behavioral differences with other animals are quantitative rather than qualitative.

In the early 1970s, Emil Menzel conducted experiments in which an ape who knew the hidden location of an attractive or frightening item (i.e., food or a toy snake) was released together with fellow apes who lacked such knowledge. The others adopted the same body language as their knowing comrade, and the concept of Theory-of-Mind was born [11],[12]. Applied to both apes and children, this concept is considered critical in relation to autism spectrum disorder. Chimpanzees were also central in the development of nonverbal mirror self-recognition tests [13], invention of the matching-to-sample paradigm that remains a staple of cognitive and neuroscience research [14], and the discovery of conflict resolution in nonhuman animals [15]. When the IOM report advised the NIH to limit the use of chimpanzees in behavioral research to studies that provide otherwise unattainable insights into normal and abnormal behavior, mental health, emotion, or cognition, it seemed to be referring to this kind of basic research as well as ongoing studies of social and prosocial behavior in apes. We should be careful, therefore, that a move to abolish biomedical research does not throw out the baby with the bathwater by also curtailing non-harmful behavioral research. In order to make the right distinctions, we need to more clearly define which procedures are ethically permissible.

My personal definition of non-invasive research on apes is simple: the sort of research I would not mind doing on human volunteers. This would include all sorts of cognitive testing, trained giving of (small) blood samples, behavioral observation, and voluntary neuroimaging. The last procedure is not yet available for apes, but likely to be developed in the near future. It is time to get a productive research program on chimpanzees off the ground without funding agencies holding its noninvasive nature against it. All they would need to do is recognize that ape research is equally constrained as ongoing human research. By continuing research that places human behavior in an evolutionary light, we would also be returning to the original rationale for bringing apes into research settings. In the 1920s, Robert M. Yerkes was the first to acquire great apes for research in the US, and his sole objective was to understand their behavior, cognition, and temperament [16].

The inevitable implication of the IOM report and the NIH's decision to momentarily halt all funding for chimpanzee-related projects is an increased focus on retirement and improved housing conditions. The report speaks of “ethologically appropriate” environments for chimpanzees, but fails to define what this means for a highly social species. Current conditions range from least to most appropriate: a) single or pair housing, b) small group housing in metal/concrete runs and primadomes, c) unroofed large outdoor corrals with grass and climbing frames, and d) multi-acre forested habitats (Figure 2). In order to offer chimpanzees optimal habitats, we should strive to move up this scale, from a→d [17]. To this end, the NIH should quantify the current housing conditions of its chimpanzees and set goals of where the captive population should be 5 to 10 years from now. The beauty of moving up this scale is that it will substantially save maintenance costs as estimated by the NIH itself [18]. It naturally costs more to clean cages every day than to house chimpanzees outdoors where the weather takes care of waste. The more spacious their housing, the more cost-efficient it will be, hence retirement under optimal conditions will be a win-win move.

**Figure 2:**
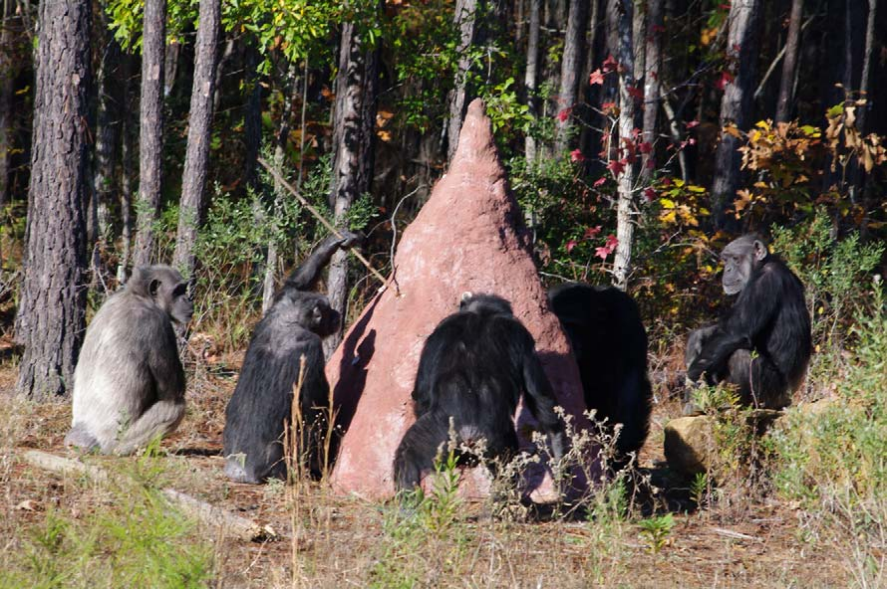
Retirement from research is to be expected for many of the chimpanzees currently at biomedical facilities. Chimp Haven, Inc. offers forested habitats, large social groups, and AAALAC-accredited care for chimpanzees retired from government sponsored projects. The sanctuary currently provides a home to over 130 chimpanzees and has almost 100 hectares on which to expand. An artificial “termite mound” (above) serves as an enrichment device, allowing chimpanzees to use tools to retrieve treats, much like their wild counterparts do to extract termites. Image credit: Chimp Haven/Amy Fultz.

The three main recommendations of the IOM report are a) put a halt to all or almost all invasive biomedical research on chimpanzees, b) a continuation of non-invasive research to evaluate human-ape similarities and differences in genetics, behavior, and neuroscience, and c) retirement and/or improved housing. The first reaction of the NIH has been to take these recommendations seriously. Being mindful of the quote from Wolfgang Goethe featured on IOM reports (“Knowing is not enough; we must apply. Willing is not enough; we must do”), this nation can expect substantial changes in its treatment of apes as a result of the overdue recognition of their special moral status.

**About the Author**

Dr. de Waal received his PhD in Biology and Zoology from Utrecht University, the Netherlands, in 1977. He completed his postdoctoral study of chimpanzees while associated with Utrecht University, in 1981, and moved the same year to the United States. He has been a National Academy of Sciences member since 2004, and a Royal Dutch Academy of Sciences member since 1993. *Time* featured him in 2007 as one of the World's One Hundred Most Influential People. He is C. H. Candler Professor of Psychology at Emory University and Director of the Living Links Center at the Yerkes National Primate Research Center.

## Abbreviations

IOM: Institute of Medicine
NIH: National Institutes of Health
